# On the Home Front: Specialised Reference Testing for Dengue in the Australasian Region

**DOI:** 10.3390/tropicalmed3030075

**Published:** 2018-07-15

**Authors:** Alyssa T. Pyke, Wendy Gunn, Carmel Taylor, Ian M. Mackay, Jamie McMahon, Lauren Jelley, Ben Waite, Fiona May

**Affiliations:** 1 Public Health Virology Laboratory, Forensic and Scientific Services, Coopers Plains, QLD 4108, Australia; Carmel.Taylor@health.qld.gov.au (C.T.); Ian.Mackay@health.qld.gov.au or Ian.Mackay@uq.edu.au (I.M.M.); Jamie.McMahon@health.qld.gov.au (J.M.); 2 Institute of Environmental Science and Research Limited, Wallaceville, 5018 Upper Hutt, New Zealand; wendy.gunn@esr.cri.nz (W.G.); Lauren.Jelley@esr.cri.nz (L.J.); Ben.Waite@esr.cri.nz (B.W.); 3 Child Health Research Centre, The University of Queensland, South Brisbane, QLD 4101, Australia; 4 Metro North Public Health Unit, Metro North Hospital and Health Service, Queensland Health, Windsor, QLD 4030, Australia; Fiona.May@health.qld.gov.au

**Keywords:** dengue, dengue virus, arbovirus, chikungunya virus, Zika virus, reference laboratory, real-time RT-PCR, ELISA, microsphere immunoassay, luminex, genotyping, whole genome sequencing

## Abstract

Reference laboratories are vital for disease control and interpreting the complexities and impact of emerging pathogens. The role of these centralized facilities extends beyond routine screening capabilities to provide rapid, specific, and accurate diagnoses, advanced data analysis, consultation services, and sophisticated disease surveillance and monitoring. Within the Australasian region, the Public Health Virology Laboratory (PHV), Forensic and Scientific Services, Department of Health, Queensland Government, Australia, and the Institute of Environmental Science and Research Limited (ESR), New Zealand (NZ) perform specialised reference testing and surveillance for dengue viruses (DENVs) and other emerging arthropod-borne viruses (arboviruses), including chikungunya virus (CHIKV) and Zika virus (ZIKV). With a focus on DENV, we review the reference testing performed by PHV (2005 to 2017) and ESR (2008 to 2017). We also describe how the evolution and expansion of reference-based methodologies and the adoption of new technologies have provided the critical elements of preparedness and early detection that complement frontline public health control efforts and limit the spread of arboviruses within Australasia.

## 1. Introduction

The recent global impact of the emergence of chikungunya (CHIKV) and Zika (ZIKV) viruses has rendered considerable and serious disease burdens throughout the tropics and subtropics. However, dengue disease remains unsurpassed as the most rapidly-emerging mosquito-borne viral disease in humans [1]. Now endemic in more than 100 countries, dengue prevalence is widespread. Regions particularly affected include Southeast Asia, the Americas, and the Western Pacific, where abundant populations of the mosquito vectors *Aedes aegypti* and *Ae. albopictus* exist, and viral transmission has been aggravated by increased human travel and trade [1]. Dengue viruses (DENVs) are classified into four antigenically distinct serotypes (DENV 1–4) [2] and are estimated to infect approximately 390 million people annually worldwide, with about 96 million (25%) showing clinical symptoms [3].

Dengue usually presents as an acute febrile illness accompanied by headache, myalgia, and arthralgia, and up to 50% of symptomatic patients develop a maculopapular rash. Constipation, diarrhoea, and respiratory symptoms are less frequently reported and hepatitis and encephalopathy are rare [4,5]. However, the risk of potentially life-threatening conditions, such as dengue hemorrhagic fever (DHF) and dengue shock syndrome (DSS), may be increased in regions where hyper-endemicity and co-circulation of more than one DENV serotype occur [4,6]. An estimated 500,000 people are hospitalized with severe dengue each year, and the incidence of mortality among these is ≈2.5% [1].

Consistent with global trends and posing substantial public health challenges, the number of reported cases of dengue within the Western Pacific continues to rise and has more than doubled from ≈200,000 in 2008 to more than 450,000 cases in 2015 [6]. In 2016, several large outbreaks and more than 375,000 suspected cases were recorded in the region, including ≈7000 cases from the Solomon Islands [1]. Dengue is predominantly an endemic disease in the Western Pacific, where its incidence is influenced by the rapid progression of socioeconomic factors, including urbanisation, and the movement of people and goods [6]. Whilst there have been substantial efforts to strengthen DENV detection and outbreak responses, limitations in medical and diagnostic capabilities, surveillance, and mosquito vector control programs still create challenges that can hinder dengue disease management in the region [6,7,8,9]. 

Indeed, ongoing transmission of DENVs and recent epidemics of CHIKV and ZIKV within the Pacific [10,11,12,13,14,15,16] have had a major impact on vulnerable, non-endemic countries, like Australia, which have populations of *Ae. aegypti* and *Ae. albopictus* mosquitoes and are at risk of periodic outbreaks initiated by viraemic travellers [7,17]. Between 1996 and 2016, the number of DENV outbreaks recorded in northern Queensland, Australia (per five year intervals) continually rose from four (1996–2000) to 32 (2011–2016) outbreaks [7], and primarily involved strains originally imported from Indonesia and Papua New Guinea (PNG) [18]. New Zealand (NZ) is considered non-endemic for arthropod-borne virus (arbovirus) disease and currently lacks *Ae. aegypti* and *Ae. albopictus* mosquitoes. Nonetheless, mosquito importation from overseas remains an ongoing risk. In a previous report describing entomological monitoring in Auckland, NZ, during 1992–1993, all live stages of *Ae. albopictus* (larval instars, pupae, and adults) were intercepted a total of three times following importation of used tires from Japan [19]. If additional factors, including environmental conditions, are also favourable at the time of mosquito introduction, further spread and establishment of susceptible mosquito species could occur. Interestingly, NZ experienced record hot temperatures in January 2018, and in the same month, mosquito surveillance demonstrated increases in the numbers of adult mosquitoes and larvae by 231% and 122%, respectively (in particular, *Ae. notoscriptus* and *Culex quinquefasciatus*), compared to the same period in January 2017 [20].

At present, global DENV outbreaks chiefly involve one or more of the four DENV serotypes, which typically circulate in human transmission cycles in urban settings [2]. However, sylvatic DENV transmission between non-human primates and arboreal *Aedes* mosquitoes remains active in West Africa, Malaysia, and Brunei, resulting in sporadic infections in humans [21,22,23,24,25,26,27]. Incidental spillover and the emergence of sylvatic DENV strains in urban transmission cycles can be facilitated by urbanization and deforestation of sylvan environments. However, as recreational and business travel continues to surge and new international attractions, including ecotourism (targeting exotic natural environments), become more popular, it is conceivable that the dispersal of rare, exotic sylvatic strains may also become more frequent, along with that of established urban strains. Viraemic travellers are capable of transporting strains thousands of kilometres into new locations, including DENV-susceptible regions. In 2014 and 2015, two viraemic patients, infected with highly divergent Brunei 2014 DENV-1 (Brun2014) and Sabah 2015 DENV-2 (Sab2015) strains, respectively, travelled from Borneo to Brisbane, Australia [21,28,29].

The cardinal elements necessary for preventing and limiting the spread of emerging DENVs and other arboviruses at the local, national, and international level include preparedness, early and rapid detection, effective and ongoing surveillance, disease management, and vector control measures. Reference laboratories are indispensable in providing specialised, tertiary (referral) level diagnostic testing, expert consultation services, and high-level containment research capabilities, otherwise unavailable in most local diagnostic laboratories [30]. Both Australia and NZ are multicultural countries, with close geographic proximity to Southeast Asia and regions of the South and Western Pacific where DENVs circulate and arbovirus epidemics are frequent. The Public Health Virology Laboratory (PHV), Department of Health, Queensland Government, Australia, and the Institute of Environmental Science and Research Limited (ESR), NZ, perform specialised reference testing for DENVs and other arboviruses of public health importance within the Australasian region. With a focus on DENV, we review the multi-faceted testing and surveillance performed by PHV (2005 to 2017) and ESR (2008 to 2017). We highlight the evolution and refinement of reference-based investigations, which are necessary to meet the ongoing challenges and greater disease intensity associated with recent arbovirus emergence, spillover, transmission, and dispersal. 

## 2. Materials and Methods

### 2.1. Ethics Statement

All methods have been performed in accordance with approved ethical guidelines. All patient samples were anonymised. This report was reviewed and approved for submission by the Queensland Health Forensic and Scientific Services Human Ethics and the Ministry of Health, NZ Health and Disability Ethics Committees.

### 2.2. Patient Samples and Diagnostic Reporting

Acute and convalescent serum samples from clinical patients suspected of DENV, CHIKV, or ZIKV infection were referred to PHV and ESR from local and international collection centres and routine diagnostic laboratories for molecular reverse transcription real-time polymerase chain reaction (RT-rtPCR), RT-PCR, and/or serological analyses. The choice of test type was mostly decided by referring clinicians and/or public health units (PHUs), who are guided by arbovirus management plans, such as the Queensland Dengue Management Plan 2005–2020 [31], but occasionally was made following consultation with PHV/ESR and further consideration of related factors, including travel history and exclusion testing, which had already been performed.

For PHV, confirmed positive results of these notifiable diseases were entered into the Queensland Health integrated Laboratory Information System (AUSLAB) and captured on the Notifiable Conditions System (NOCS) database, which is accessible to PHUs, the Communicable Diseases Branch at Queensland Health, and other Queensland and Australian agencies. Urgent and important case results were notified immediately to PHUs by telephone and/or email. PHV also performed testing on samples from people who did not travel to Australia, but were referred directly from other overseas medical centres or laboratories. Similarly, confirmed positive results from ESR were entered into the STARLiMs Laboratory Information System, which automatically generates notifications for NZ PHUs.

PHUs in Queensland and NZ are responsible for communicating with positively-identified infected patients and facilitating follow-up specimen collections, collation of travel histories, and relevant clinical data. The PHUs further coordinate disease management strategies, including mosquito vector control programs (along with other government agencies) and contact tracing. Queensland is receptive to local DENV, CHIKV, and ZIKV transmission due to established populations of *Ae. aegypti and Ae. albopictus* mosquitoes. Following an urgent PHU request, PHV endeavour to assess and report findings within 24–48 h of receipt of samples. Disease management is activated immediately by PHUs following positive case notification. Local transmission is suspected if the positive case has not travelled (one locally-acquired case constitutes an outbreak), and further control strategies, including vector control, are implemented immediately (reviewed in [31]).

### 2.3. Testing Protocols

#### 2.3.1. PHV Overview

For the diagnosis of suspected acute DENV, CHIKV, or ZIKV infections, serum specimens collected during the viraemic phase (usually between day zero and day five post-onset of symptoms, or longer for urine samples in the case of ZIKV infections [32]) were tested by both molecular and serological diagnostic platforms. Whilst optimal timing of specimen collection can vary between detection methods and sample types, molecular viral RNA detection is usually more sensitive during the zero- to five-day period after symptom onset. Serological detection of patient IgM and IgG antibodies is generally possible later in the infection, commencing approximately two and six days post-infection, respectively [31]. A positive RT-rtPCR/RT-PCR result confirms acute infection whereas the absence of a detection of viral RNA cannot be used to exclude infection. Thus, PHV perform IgM/IgG serology on all sera submitted for molecular testing. Conversely, molecular testing on sera submitted for serological analysis is only performed on request from the referring clinician or PHU, or following consultation with PHV. This may be appropriate in the case of secondary infections where patients are clinically suspected of an arboviral infection and are negative for IgM antibodies or have produced specific IgM antibodies to a previously infecting (and different) arbovirus due to an anamnestic response to the current infection.

PHV report initial IgM/IgG findings on single specimens, but a second convalescent-phase sample collected 10–14 days after the first is requested on all positive and negative IgM/IgG samples to confirm or exclude infection. This facilitates parallel testing of acute and convalescent-phase samples aimed at the detection of IgM/IgG antibody seroconversions or measurable rise or fall in specific antibody titers, both of which are optimal for specific viral diagnosis. A summary of the testing algorithms for acute DENV infections is provided in Supplementary Figure S1. The response rate of clinicians in providing a second patient sample varies, depending on the viral infection suspected or if initiated by a PHU. For ZIKV infections, provision of a convalescent-phase sample was reported in 60.6% of cases [33]; however, PHV usually only receive convalescent-phase samples in <30% of cases.

PHV employ a large number of specific molecular RT-rtPCR/RT-PCR assays for the diagnosis of acute infections caused by arboviruses and combine these analyses with serological investigations. Similar to molecular methodologies, non-structural protein 1 (NS1) ELISA assays target the viral organism and can be useful for the rapid detection of acute viral illnesses. NS1 is optimally detectable from 1–2 days and up to nine days post-infection. Whilst some local hospital and private pathology laboratories use commercially available DENV and ZIKV NS1 strip/ELISA kits, they do not have access to or cannot employ the extensive suite of molecular assays utilized by PHV. As a reference laboratory, PHV maintains assays that are not in common use and this greatly assists further scrutiny and confirmation of positives that have been screened by alternate commercial assays. This includes multiple RT-rtPCR/RT-PCR assays for DENV and ZIKV and one for CHIKV (Table 1) and flavivirus ELISA/MIA protocols employing whole virus antigens made from 12 different arboviruses [33] (PHV ELISA Protocols, Supplementary Data).

##### RT-rtPCR

Viral RNA was extracted from 140 µL of acute-phase sera (or urine samples for ZIKV infections) using the QIAamp viral RNA Kit [36], or extracted from 200 µL of sample using the Qiagen BioRobot Universal System or QIAamp Virus BioRobot MDx Kit (Qiagen, Clifton Hill, Australia) [37]. Between 2005–2017, samples submitted for DENV RT-rtPCR were first screened using a pan-DENV 1–4 TaqMan real-time RT-rtPCR assay (DENUT) [38], and, if positive, confirmed by repeat testing. Positive DENUT samples were then analysed to determine the infecting serotype using specific DENV 1, 3 and 4 TaqMan RT-rtPCR assays [39] and DENV-2 RT-rtPCR assays [40,41], or a hemi-nested DENV 1–4 RT-rtPCR [34]. By 2018, new DENV TaqMan RT-rtPCR assays, namely, pan-DENV 1–4 (DU5 MGB2017), DENV-1 TM2017, DENV-2 MGB, DENV-3 TM2017, and DENV-4 TM2017, were introduced by PHV and have superseded the previously used DENUT and DENV 1–4 TaqMan RT-rtPCR assays (Supplementary Table S1). These DENV assays were designed to target 3′ untranslated regions (3′UTRs), which were more conserved within the respective DENV 1–4 serotypes, and were used to maximize detection of the large number of circulating DENV strains.

The new 3′UTR RT-rtPCR DENV assays were optimised and validated. Briefly, sensitivities of the new 3′UTR assays were compared with any previous assay version using 50 positive RNA patient extracts (where available) from each DENV 1–4 serotype. Specificity was determined by screening 100 previously negative RNA patient extracts from patients following set criteria. This included samples from a range of specimen types and samples from patients who had been infected with a different arbovirus (other than DENV) or had similar clinical symptoms. The new 3′UTR assays demonstrated 98–100% sensitivity and specificity compared to the respective DENV 1–4 RT-rtPCR assays previously used.

For CHIKV and ZIKV RT-rtPCR analysis, RNA was extracted from serum samples (and/or urine for ZIKV) as described above. The CHIKV (targeting the envelope 1 (E1) gene) and ZIKV (targeting the envelope (E) and NS1 genes) oligonucleotide primers and probes were as previously reported [11,35].

The following RT-rtPCR reagents and cycling conditions were common to all DENV, CHIKV, and ZIKV molecular assays. Each 20 µL single-tube, Superscript^®^ III Platinum^®^ one-step qRT-PCR (Invitrogen, Carlsbad, CA, USA) reaction included 0.4 µL Superscript™ III RT/Platinum^®^ Taq mix, 10.0 µL of 2× reaction mix, oligonucleotide primers and probes (concentrations listed in Supplementary Table S1), 50 nM ROX reference dye, and 5 µL of extracted viral RNA, water (for no template control; NTC), or positive control template. Amplification conditions included one cycle at 50 °C for 5 min, one cycle at 95 °C for 2 min, and 40 cycles at 95 °C for 3 s and 60 °C for 30 s.

##### DEN 1–4 RT-PCR

The DENV 1–4 hemi-nested RT-PCR designed in the capsid-premembrane (C-prM) genomic region, has been described previously [34]. Oligonucleotide primer sequences were as reported, with the exception of TS1 and TS2, which were modified to TS1-bis (5′-CGTCTCAGTGATCCGGGGRC-3′) and TS2-B (5′-AAYGCCACMAGGGCCATGAACA-3′), respectively. Nucleic acid amplification was performed using the Superscript III/Platinum Taq High Fidelity One-Step RT-PCR System (Invitrogen, Carlsbad, CA, USA) according to the manufacturer’s instructions. First-round amplification conditions included one cycle at 55 °C for 15 min, one cycle at 94 °C for 2 min, and 35 cycles at 94 °C for 15 s, 55 °C for 30 s and 68 °C for 60 s. First-round amplification products were diluted 1:100 in DNase, RNase free water (Sigma-Aldrich, Castle Hill, Australia) before 2 µL of the diluted product was added to round two PCR mixes and amplified in a total of 20 µL using the QIAGEN Fast Cycling PCR Kit (Qiagen, Clifton Hill, Australia) as described by the manufacturer. Second round amplification conditions included one cycle at 95 °C for 5 min, 35 cycles at 96 °C for 5 s, 55 °C for 5 s, and 68 °C for 18s, and a final extension at 72 °C for 1 min. DNA amplification products were visualized on an agarose gel or analysed using the QIAxcel Advanced System (Qiagen, Clifton Hill, Australia) according to the manufacturer’s instructions.

##### Virus Culture

A selection of DENV 1–4 RT-rtPCR/RT-PCR positive serum samples representing DENVs from varied geographical locations that were imported into Australia or were locally transmitted in Queensland between 2005–2017 were chosen for viral culture. The selection of samples for viral culture depended on a number of factors, including unusual clinical presentation, travel history, date of sample collection, involvement in local transmission and newly identified transmission sites, surveillance for endemicity, and any other unusual diagnostic or genetic findings. Serum samples that had relatively low threshold cycle numbers (*C*_t_) of approximately ≤30 were optimal candidates for viral isolation. To recover DENV isolates, the acute phase serum of selected DENV 1–4 RT-rtPCR/RT-PCR positive patients were inoculated onto confluent monolayers of *Ae. albopictus* C6/36 cells (ATCC, CRL-1660) and passaged up to three times. Cultures were then assessed for specific DENV 1–4 infection using an immunofluorescence assay (IFA) and pan-flavivirus, pan-DENV, and specific DENV 1–4 monoclonal antibodies as previously described [21,42]. Similarly, CHIKV isolates from CHIKV RT-rtPCR positive samples were recovered in C6/36 cells and confirmed by IFA using pan-alphavirus 2A2C [43] and specific anti-CHIKV 3A2 [44] monoclonal antibodies. For ZIKV cultures, isolations were first attempted by inoculating C6/36 cells and African green monkey kidney (Vero) cells (ATCC, CCL-81). However, no successful isolations were recovered using conventional cell culture and inoculation of 1–2 day old Swiss outbred suckling mice was then used successfully [12].

##### Molecular Genotyping

Nucleotide sequencing and phylogenetic analysis of complete E genes were performed as previously described [7,17]. Next generation whole genome sequencing (WGS) of complete DENV, CHIKV, and ZIKV genomes were performed as previously described [28,45] and phylogenetic analysis of the complete coding region of CHIKV genomes was performed as previously reported [46].

##### Serology

PHV screen sera for anti-flavivirus IgM and IgG antibodies using in-house flavivirus ELISAs (IgM; MAC-ELISA, IgG; FLG-ELISA, Supplementary data). Samples that were IgM positive were further analysed for specific anti-flavivirus (including DENV 1–4) IgM antibodies using a flavivirus typing ELISA (FLTYP) [47] (2005–2013) or FLMIA [33] (2013–2017).

#### 2.3.2. ESR Overview

Serological diagnostics were performed at ESR using commercially-available IgG and IgM kits according to the manufacturers’ instructions. The DENV IgM/IgG ELISA (Panbio, Alere, Australia), the DENV NS1 antigen ELISA (Bio-Rad, Platelia, France), and anti-CHIKV indirect IFA (Euroimmun, Lübeck, Germany) assays have been performed since 2008, 2009, and 2015, respectively. The ZIKV ELISA (Euroimmun, Lübeck, Germany) was introduced in 2016.

The DENV NS1 assay can provide a rapid, relatively cheap confirmatory diagnosis as it is based on viral antigen detection. However, in samples with a low viral load, sensitivity of the NS1 assay may be limited as no further amplification of the viral antigen occurs. In contrast, RT-PCR based methods can facilitate rapid viral RNA amplification many magnitudes greater than the initial RNA levels present in the sample. In 2017, ESR began using the CDC Trioplex molecular assay, a multiplexed RT-rtPCR, as a molecular screening assay for DENV, CHIKV, and ZIKV (CDC, Atlanta, GA, USA) as recommended by the manufacturer. Positive DENV samples were further confirmed and serotyped using singleplex RT-rtPCR DENV 1–4 assays as previously described [48]. Samples that were positive by the Trioplex assay for CHIKV and ZIKV were also confirmed using singleplex CHIKV [49] and ZIKV [50] assays.

As described above for PHV (Section 2.3.1), ESR also request a second convalescent-phase sample to be collected following analysis of the acute-phase sample. ESR request the second sample to be taken 7–21 days (optimally 14 days) after the first bleed. Convalescent-phase sampling is requested when acute sera are positive by RT-PCR, DENV NS1 ELISA, IgM positive by DENV, ZIKV, or CHIKV ELISA, or are IgM negative and are clinically suspected for arboviral disease or secondary infections. ESR currently receives convalescent-phase samples in approximately 50% of cases.

## 3. Results

### 3.1. Multi-Faceted Reference-Based Methods

**Figure 1:**
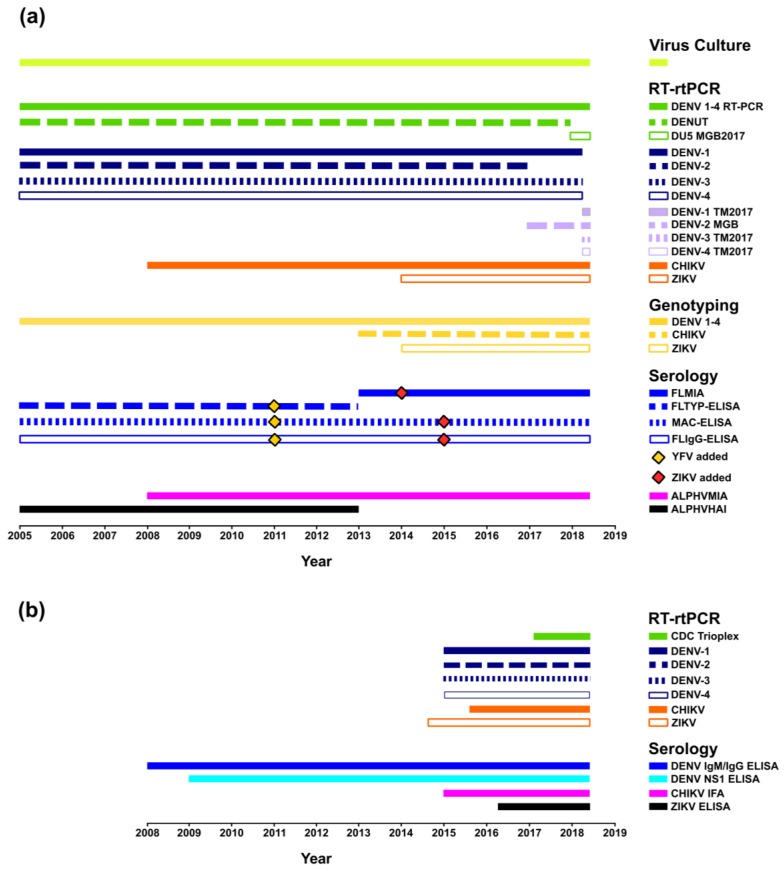
Reference DENV testing performed by PHV and the Institute of Environmental Science and Research Limited (ESR) (https://doi.org/10.6084/m9.figshare.6164183.v1). (**a**) Timeline summarising the virus culture, molecular, and serological reference tests performed by PHV (2005 until present), including the addition of new DENV in-house reverse transcription real-time polymerase chain reaction (RT-rtPCR) TaqMan assays (pan DENV 1–4 (DU5 MGB2017); DENV-1 TM2017; DENV-2 MGB; DENV-3 TM2017; DENV-4 TM2017). Yellow fever virus (YFV) and Zika virus (ZIKV) antigens were added to the serological platforms in 2011 and 2014–2015 [33], respectively, and chikungunya virus (CHIKV) antigens were included in the alphavirus hemagglutination inhibition (ALPHVHAI) and alphavirus microsphere immunoassay (ALPHVMIA) platforms; (**b**) timeline summarizing the reference testing performed by ESR (2008 until present), including the introduction of real-time TaqMan RT-rtPCR assays for DENV 1–4, CHIKV, and ZIKV by 2016 and a multiplex DENV 1–4 TaqMan RT-rtPCR assay (CDC Trioplex) in 2017. Serological assays for CHIKV (CHIKV immunofluorescence assay (IFA)) and ZIKV (Euroimmun, Lübeck, Germany) were introduced in 2015 and 2016, respectively.

Within the last 20 years, several medically important arboviruses, including DENV, CHIKV, and ZIKV, have expanded their geographical range into new regions, resulting in autochthonous transmission and large-scale epidemics [3,51,52,53]. Both PHV and ESR laboratories have been highly tuned to the global emergence of these pathogens and the influx of viraemic travellers to Australia and NZ, and this consequently, created the impetus for ensuring the availability of rapid, sensitive, and specific assays. Foresight and preparedness further accelerated the introduction of new platforms and allowed the refinement of testing algorithms and technologies by PHV and ESR. A review of the multi-faceted reference diagnostic and surveillance methods for DENV, CHIKV, and ZIKV employed by PHV (2005–2017) and ESR (2008–2017), including the development of improved and more sensitive platforms, is graphically represented in the timelines shown in Figure 1.

PHV has provided viral culture, RT-rtPCR/RT-PCR, nucleotide sequencing (initially subgenomic, now augmented by whole genome methods), molecular genotyping (phylogenetic evolutionary characterisation of DENV strains), and specific serological typing for DENV 1–4 and other arboviruses for more than 13 years. A more detailed description of these methodologies and findings are provided below. In 2005–2006, large-scale CHIKV epidemics in the Indian Ocean prompted the development of new diagnostic assays. By 2008, PHV had introduced in-house, CHIKV RT-rtPCR [35], molecular genotyping, and serological assays. These assays were available prior to the Pacific CHIKV outbreaks in New Caledonia (2011) [54] and PNG (2012) [16], and the epidemics in the Americas (2013) [55]. Similarly, PHV responded to the wider dissemination of ZIKV in the Pacific, which began in French Polynesia in 2013 [56], and, in 2014, described the first specific real-time ZIKV RT-rtPCR assays available in Australia [11]. In the same year, PHV also commenced in-house serological screening for ZIKV IgM antibody detection. Thus, by 2015, when explosive ZIKV outbreaks commenced in Brazil and later intensified throughout the Americas [57], PHV was equipped to monitor and provide laboratory assessment of viraemic travellers suspected of ZIKV infection.

ESR has provided DENV serological testing (Panbio, Alere, Australia) since 2008 and introduced a CHIKV immunofluorescence assay (IFA) (Euroimmun, Lübeck, Germany) and a ZIKV enzyme-linked immunosorbent assay (ELISA) (Euroimmun, Lübeck, Germany) in 2015 and 2016, respectively. Molecular RT-rtPCR diagnostic platforms were introduced for DENV 1–4 (2015), CHIKV (2015), and ZIKV (2014) in response to increased co-circulation of these viruses in the Pacific region combined with the escalating number of imported cases. ESR also implemented the Trioplex RT-rtPCR (Centers for Disease Control and Prevention (CDC), Atlanta, GA, USA) in 2017 as a multiplex assay to screen for DENV, CHIKV, and ZIKV.

Recent global ZIKV outbreaks and co-circulation of this pathogen in many DENV endemic regions have complicated arbovirus diagnostic testing. Immune cross-reactivity of patient antibodies in serological assays can compromise the specific diagnosis of flavivirus infections, in particular, those caused by DENV and ZIKV [58]. The multi-faceted utilization of molecular and serological platforms employed by both PHV and ESR is highly desirable and advantageous for the specific diagnosis of acute-phase samples compared to the use of conventional ELISA alone. As recently described [33], PHV used a combination of RT-rtPCR/RT-PCR and MIA serological platforms to assess a panel of sera from patients suspected of ZIKV infection. In that study, specific ZIKV infection could be confirmed in 99 of 101 suspected cases and included the differentiation between primary and secondary infections [33].

### 3.2. Confirmed Cases

#### 3.2.1. PHV

**Figure 2:**
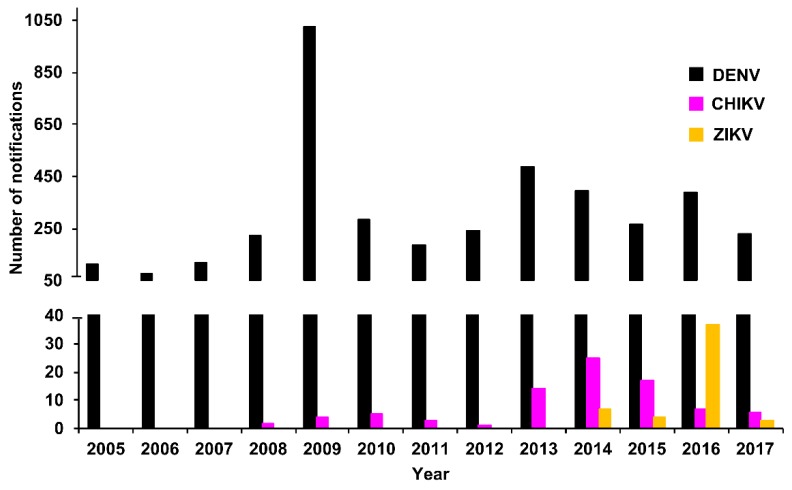
Notified DENV cases by year, Queensland, 2005–2017. Number of DENV, CHIKV, and ZIKV case notifications reported annually in Queensland, 2005–2017 (DENV and CHIKV, National Notifiable Diseases Surveillance System, Department of Health, Australian Government and ZIKV [59]). Notifications of CHIKV and ZIKV disease commenced in 2008 and 2014, respectively.

We collated the DENV case notifications (laboratory-confirmed positive cases) and compared these with notifications of two other prominent arboviruses, CHIKV and ZIKV, which began in 2008 and 2014, respectively. The total number of DENV, CHIKV, and ZIKV notifications for Queensland per year for the period 2005–2017 (DENV and CHIKV, National Notifiable Diseases Surveillance System, Australian Government, Department of Health, and ZIKV, [59]) is summarised in Figure 2. The total number and mean notifications per year reported per virus between 2005 and 2017 are summarised in Table 2. Of note, elevated DENV (2009) cases corresponded to an explosive outbreak of DENV-3 in Cairns, Queensland, Australia in 2008–2009 [60]. Similarly, elevated CHIKV (2013–2015) and ZIKV (2014–2016) cases were largely due to a CHIKV outbreak in PNG (2012–2013) [16], increased CHIKV transmission in the Pacific and introduction into the Americas [61], and extensive ZIKV incursions in the Pacific and the Americas [52,56].

##### RT-rtPCR and RT-PCR

As with all oligonucleotide PCR formats, RT-rtPCR assays can be prone to primer/probe sequence mismatches with the target viral RNA template, reducing sensitivity or preventing detection. As described above in Table 1 and Figure 1, PHV maintain a suite of molecular assays to maximize detection of the large number of globally circulating DENV strains.

For the period, 2005–2017, PHV largely performed RT-rtPCR for the molecular diagnosis of patients with acute DENV 1–4 infections. A hemi-nested DENV 1–4 RT-PCR [34] was also used to assist molecular typing of DENV RNA-positive samples. In addition to samples collected locally in Australia, PHV also tested samples that were directly referred from overseas medical centres or laboratories. Although these were collected from cases that did not travel to Queensland, we have reported the corresponding positive sample data from these patients to further demonstrate the prevalence of arboviruses in the region and highlight the potential disease threat posed by people who may travel from these locations to Australia. 

**Figure 3:**
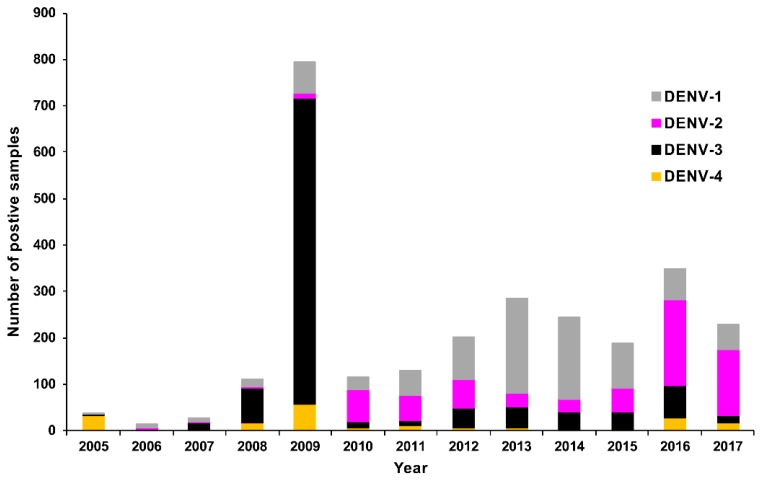
Summary of confirmed DENV 1–4 RT-rtPCR/RT-PCR-positive samples reported by PHV, Queensland between 2005 and 2017 by year and serotype.

The number of confirmed DENV RT-rtPCR/RT-PCR-positive samples reported by PHV between 2005 and 2017 is summarised in Figure 3 by serotype. The total number of positive samples for the period was 2729 (DENV-1, 871; DENV-2, 636; DENV-3, 1038; DENV-4, 184). The serotype with the highest total number of positive samples was DENV-3. This was largely due to the rapid autochthonous transmission that occurred during the explosive DENV-3 epidemic in Cairns, Queensland, between 2008–2009, resulting in 931 cases and one death [60]. Between 2012 and 2015, DENV-1 was the most prevalent serotype, with 568 positive samples recorded. There were 20 DENV outbreaks in northern Queensland during this period, including 16 (80.0%; 489 confirmed cases) caused by DENV-1 [63]. In 2016 and 2017, DENV-2 was the dominant serotype reported (325 positive samples) and was responsible for 7 (53.8%; 33 confirmed cases) of the 13 outbreaks recorded in northern Queensland [63]. Also during 2016–2017, several large dengue outbreaks were recorded in the Pacific, including reports of DENV-2 transmission in Vanuatu, American Samoa, Fiji, and New Caledonia [64]. Similarly, PHV reported DENV-2 RT-rtPCR/RT-PCR-positive cases among residents from the Solomon Islands, Vanuatu, Fiji, Nauru, and Samoa.

Dual infections were identified by RT-rtPCR in two cases (both resulting from DENV-1 and DENV-2 co-infections). Each patient had travelled to an endemic DENV region, visiting Bali (2014) and Malaysia (2015), respectively.

**Figure 4:**
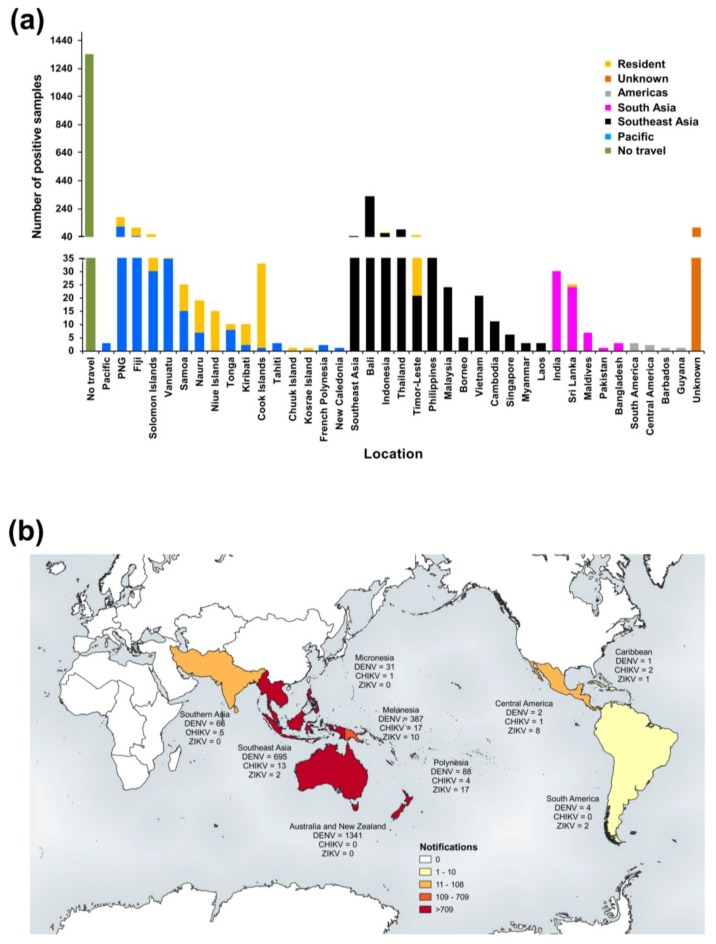
RT-rtPCR/RT-PCR-positive samples reported by PHV, Queensland between 2005 and 2017 by location (region travelled from or residence). Subregional classifications were taken from the United Nations Statistics Division at https://unstats.un.org/unsd/methodology/m49/. (**a**) Number of DENV RT-rtPCR/RT-PCR-positive samples. Allocations to specific locations or broader subregional classifications (which exclude specific location data) were dependent on the travel information available. Patients with no travel (no overseas travel outside Australia) and patients who did not travel to Australia (resident of that location) are shown separately, including cases from the Pacific Islands, Niue, Chuuk, and Kosrae. Corresponding summaries of CHIKV and ZIKV RT-rtPCR-positive samples based on location are shown in Supplementary Figure S2B,D, respectively); (**b**) Choropleth map of positive DENV, CHIKV, and ZIKV RT-rtPCR/RT-PCR samples (https://doi.org/10.6084/m9.figshare.6220481.v2) based on subregional classifications. Where a sample was positive for more than one virus (e.g., DENV-1 and DENV-2), the sample was only included in the map once (*n* = 2). Three additional positive samples (two from the Pacific and one from the South Pacific) could not be allocated to the map subregions and were not included. Map was created using QGIS software version 3.0.1.

The number of positive DENV RT-rtPCR/RT-PCR samples reported by PHV based on travel status (*n* = 2729) is shown in Figure 4a. The largest number of these positive samples (*n* = 1341, 49.1%) were collected from people who had not travelled overseas during their exposure period and were locally infected in Queensland. The most frequently reported regions for overseas-acquired infection among travellers (*n* = 1106) were Southeast Asia (*n* = 662, 59.9%) and the Pacific (*n* = 263, 23.8%). Where specific location data were available, the largest number of positive samples among travellers came from Indonesia (*n* = 391, 35.4%) and PNG (*n* = 113, 10.2%). Of the Indonesian travellers, 326 (83.4%) specifically named Bali as the location from which they had travelled.

For comparison, the number of CHIKV (2008–2017) and ZIKV (2014–2017) RT-rtPCR positives reported by PHV is summarised in Supplementary Figure S2. There was a total of 43 positive CHIKV RT-rtPCR samples. Of these, most were collected from travellers (visitors and local returning residents, *n* = 28) and the remaining positives (*n* = 15) were from patients who resided overseas. Most of the 28 travellers had acquired their infection in Southeast Asia (*n* = 12, 42.9%) or the Pacific (*n* = 8, 28.6%). From the 43 positive samples, 17 (39.5%) were from PNG in 2013, likely resulting from the 2012–2013 CHIKV PNG outbreak [16], and included samples from three travellers and 14 PNG residents. There was one positive sample from a Timor-Leste resident, which had been submitted to PHV in 2009.

There were 43 ZIKV RT-rtPCR-positive samples reported by PHV between 2014 and 2017. Most visitors and local returning travellers (*n* = 37) were infected in the Pacific (*n* = 21, 56.8%), of which 8 (21.6%) were infected in the Cook Islands. A further 11 of the total 43 positive samples (25.6%) were from patients who had travelled from the Americas and, of these 11 positives, 6 (54.5%) positive samples were from travellers from Mexico.

To summarise the collective numbers of DENV, CHIKV, and ZIKV RT-rtPCR/RT-PCR-positive samples by patient travel status, we created a choropleth map based on global subregional groups (Figure 4b). Regions in close geographical proximity to Australia were among those representing the largest number of positive samples, but PHV also identified cases that had travelled significant distances to Australia (including travellers from Southern Asia and the Americas).

##### DENV Serology

PHV performs serological testing for acute arbovirus infections using rapid, semi-automated, and sensitive in-house-developed IgG and IgM enzyme-linked immunosorbent assay (ELISA) and Luminex^®^ microsphere immunoassay (MIA) platforms. A more recent development, the MIA assay was introduced in 2013 to increase diagnostic capability and replace more labour-intense and less sensitive hemagglutination inhibition (HAI) and ELISA typing assays. Further, MIA technology has afforded the advantages of multi-analyte profiling in a single reaction well, allowing an individual serum to be analysed against several viral antigens simultaneously and the fluorescence intensity outputs to be measured and compared in real-time [65]. PHV introduced alphavirus (ALPHVMIA) and flavivirus (FLMIA) assays in 2008 and 2013 [33], respectively. These multi-target, rapid, and reproducible techniques support clinical decision-making for these viruses, which may have short windows of viremia. Further, these platforms provide additional interrogation of serological screening results where interpretation is confounded by the potential for multiple related viral exposures in the regions of travel.

**Figure 5:**
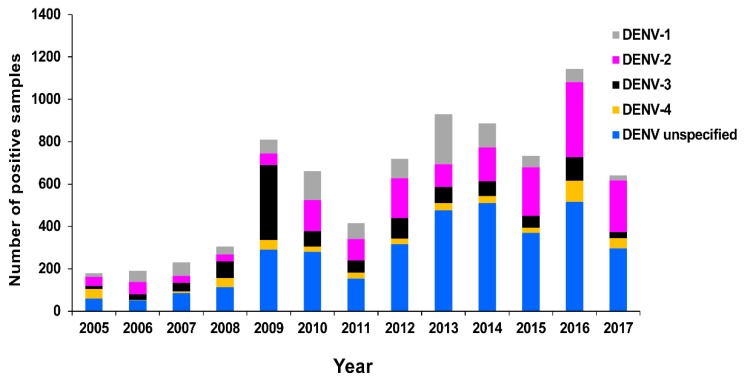
Number of DENV-positive IgM samples reported by PHV, 2005–2017, by year and serotype.

PHV currently screens sera for flavivirus infections using IgG (FLIgG-ELISA) and IgM (MAC-ELISA) [47] assays (Supplementary data, PHV ELISA Protocols). Samples are assessed for reactivity to a pool of flavivirus antigens, including DENV 1–4 and ZIKV. Positive IgM samples are further confirmed and typed using individual flavivirus antigens in an in-house IgM FLMIA [33]. Sera submitted for DENV RT-rtPCR are also tested by FLIgG-ELISA and MAC-ELISA to further characterise acute infections and minimise the misinterpretation of negative RT-rtPCR findings from samples collected outside the viraemic infection phase. As described above (Section 2.3.1), PHV routinely request a second convalescent-phase sample to be collected 10–14 days after the acute-phase sample to facilitate parallel testing between the paired sera. For the period, 2005–2017, PHV reported 7840 DENV IgM-positive samples (summarised in Figure 5). More samples were positive for DENV-2 IgM (*n* = 1749, 22.3%) compared to any other serotype (DENV-1, *n* = 1025 [13.1%]; DENV-3, *n* = 1085 [13.8%]; DENV-4, *n* = 461 [5.9%]). A further 3520 DENV IgM-positive samples (DENV unspecified) could not be typed due to cross-reacting antibodies. In 2009, 355 DENV-3 IgM positive samples were reported, coinciding with the large local DENV-3 outbreak that occurred in Cairns, Queensland in 2008–2009 [60].

##### Virus Culture

Rapid and specific RT-rtPCR assays have largely superseded traditional and more laborious arbovirus culture methods in routine diagnostics. Molecular PCR platforms can also detect RNA from dead/neutralised virus particles or partial RNA molecules in samples. Several factors, including specimen transport, suboptimal storage temperature/conditions, the timing of collection, and presence of neutralising antibodies, can negatively impact on the success rate of virus culture. Despite these challenges, PHV continues to isolate representative viruses from varied geographical locations directly from clinical samples. Clinical virus isolates are essential to public health investigations, surveillance, and characterisation of viruses. Contemporary and historical virus strains are useful for WGS analysis and molecular genotyping, mosquito vector competence experiments, production of in-house diagnostic serology antigens, molecular assay development, and for research of virus pathogenesis and replication.

**Figure 6:**
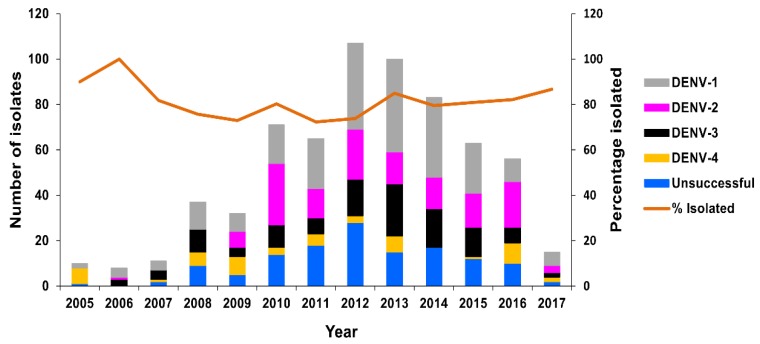
Number of DENV isolates obtained, PHV, 2005–2017. The number of RT-rtPCR/RT-PCR-positive samples from which DENV isolates were obtained (grey, pink, black, and yellow bars) or not obtained (blue bar) is highlighted per year. The average percentage of successfully-isolated viruses per year is shown as a line.

Between 2005 and 2017, virus isolation was attempted from 658 DENV RT-rtPCR-positive serum samples, resulting in 525 (79.8%) successful DENV isolations, including 221 DENV-1, 136 DENV-2, 116 DENV-3, and 52 DENV-4 isolates. Isolation recovery varied between 72.3% and 100% per year, with an average of 79.2% per year (Figure 6).

Among these, PHV was the first to diagnose and isolate from clinical patients two highly-divergent sylvatic strains, DENV-1 Brun2014 [21] and DENV-2 Sab2015 [28,29], imported from Borneo in 2014 and 2015, respectively. Further, PHV has demonstrated that Brun2014 can disseminate in *Ae. aegypti* mosquitoes at rates not significantly different from an urban DENV-1 strain, and, therefore, could potentially facilitate an urban outbreak, particularly if *Ae. aegypti* mosquito numbers are high [21].

Representative CHIKV isolates were also obtained from CHIKV RT-rtPCR-positive serum samples. Between 2008 and 2017, 15 CHIKV isolates were successfully recovered from a total of 22 RT-rtPCR-positive samples. The percentage of viral recovery varied between 0 and 100% per year, with an average of 78.5% per year (Supplementary Figure S3).

PHV also attempted to isolate ZIKV from 19 RT-rtPCR-positive clinical samples between 2015 and 2016 using conventional cell culture in C6/36 cells (as used for DENV and CHIKV) and mammalian Vero cells. No isolates were recovered from direct inoculation of clinical samples onto cells. Inoculation of suckling mouse brains was then attempted for eight of the RT-rtPCR-positive samples. One isolate was obtained from the serum of a traveller to Tonga in 2016. This was the first successful isolation of ZIKV from a human clinical sample in Australia and the first globally reported Tongan isolate [12]. The TS17-2016 Tongan isolate provided an essential representative strain of the currently circulating Asian ZIKV lineage implicated in global epidemics and cases of Guillain-Barré syndrome and microcephaly in infants. The TS17-2016 strain was important for the validation of molecular and serological assays and mosquito vector competence experiments using local *Ae. aegypti* from northern Queensland [66].

##### Molecular Genotyping

To assess the risk of locally transmitted DENVs becoming established in Queensland, and to determine the genetic and evolutionary relatedness of these and other imported DENVs with globally circulating DENV 1–4 strains, PHV has been performing DENV nucleotide sequencing and molecular genotyping for more than 13 years. The primary initiative for these investigations has been to assist public health management of outbreaks by identifying outbreak origins, confirming epidemiological links, monitoring strain movement, and assessing potential viral establishment and endemicity. Molecular genotyping and phylogenetic analysis of imported and circulating strains has also elucidated the genetic diversity of DENVs in the Australasian region [7,17], and identified importation of rare, highly divergent DENVs [21,28,29]. Research is ongoing to understand whether these genetically diverse viruses replicate, transmit, or cause disease differently from other urban DENV strains. Given the continued introduction of DENVs and limited availability of effective vaccines, virus surveillance remains critical for the deployment of vector control resources into areas where they will have the greatest impact.

Within the last three decades (1990–2017), there have been at least 73 DENV outbreaks recorded in northern Queensland [18] (Supplementary Figure S4), which is prone to DENV, CHIKV, and ZIKV outbreaks due to established populations of both *Ae. aegypti* and *Ae. albopictus* mosquitoes [67,68]. Recently, we reported that Southeast Asia was the major source of Queensland DENV outbreak strains (40 outbreaks, 54.8%), which have involved all four serotypes [18]. By country, Indonesia was the largest contributor of these imported outbreak strains (17 outbreaks, 23.3%), followed by PNG (15 outbreaks, 20.5%) [18]. Indeed, PNG is also an important source of DENVs, which has contributed to north Queensland outbreaks. Recently, and for the first time, we demonstrated molecular evidence of DENV 1–3 hyperendemicity in PNG, and the evolution of new genetic lineages [7].

The introduction of WGS has revolutionized viral genomic analysis, proving useful for the discovery and genetic characterisation of arboviruses [69]. Whilst WGS is not yet practical in terms of cost and turnaround times for routine screening of diagnostic specimens, PHV have utilized WGS to sequence genomes of rare and emerging DENV, CHIKV [21,28,46], and ZIKV pathogens. Of note, PHV generated the first globally reported whole genome sequence of a highly divergent DENV strain (GenBank Accession number KR919820) and the first whole genome sequences of CHIKV strains from PNG and Kiribati (GenBank Accession numbers MF773569 and MF773562, respectively) and a ZIKV strain from Tonga (TS17-2016, GenBank Accession number KX806557). Further, WGS and phylogenetic analysis of viruses isolated from 11 CHIKV cases imported into Queensland between 2010 and 2017 demonstrated new insights into the emergence and spread of CHIKV from Southeast Asia. These findings indicated that CHIKV expansion into the Pacific and Americas likely involved multiple introductions of strains from Southeast Asia [46].

#### 3.2.2. New Zealand

The total number and mean number of notifications per year reported per virus between 2008 and 2017 are summarised and compared with Queensland notifications in Table 2. Total numbers of confirmed cases reported per virus by location are summarised in Supplementary Table S2. Of the positive DENV cases, a total of six DENV-1, 44 DENV-2, 31 DENV-3, six DENV-4, and 711 DENV unspecified notifications were reported. The majority of cases reported by location were infected in the Pacific region and resulted in a total of 408 DENV, 69 CHIKV, and 90 ZIKV notifications. Most DENV infections by single location (*n* = 798) were in travellers from Indonesia (*n* = 142, 17.8%). Similarly, the largest number of reported CHIKV (*n* = 87) and ZIKV (*n* = 98) infected travellers were from Samoa (*n* = 39, 44.8%) and Tonga (*n* = 49, 50%), respectively. ESR also reported one additional ZIKV case with no travel history outside of NZ, having acquired the infection locally via sexual transmission [62]. This case was excluded from the analysis.

**Figure 7:**
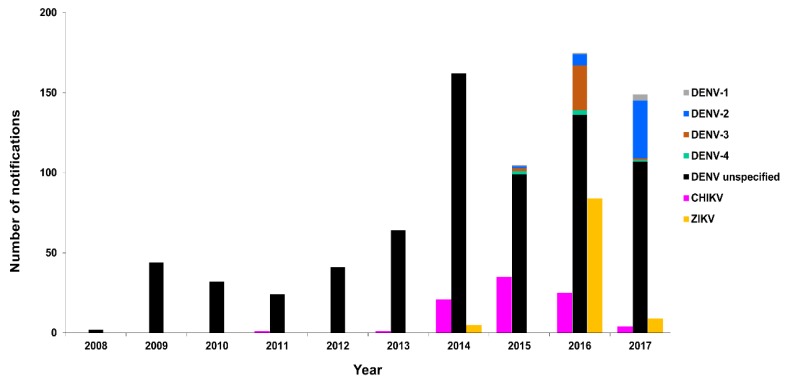
Notified cases by year, New Zealand (NZ), 2008–2017. Number of notified confirmed DENV 1–4, CHIKV, and ZIKV cases reported in NZ by year, 2008–2017 [70].

**Figure 8:**
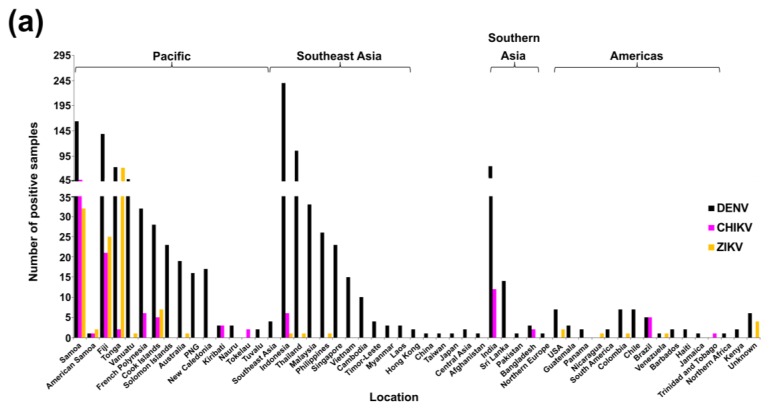

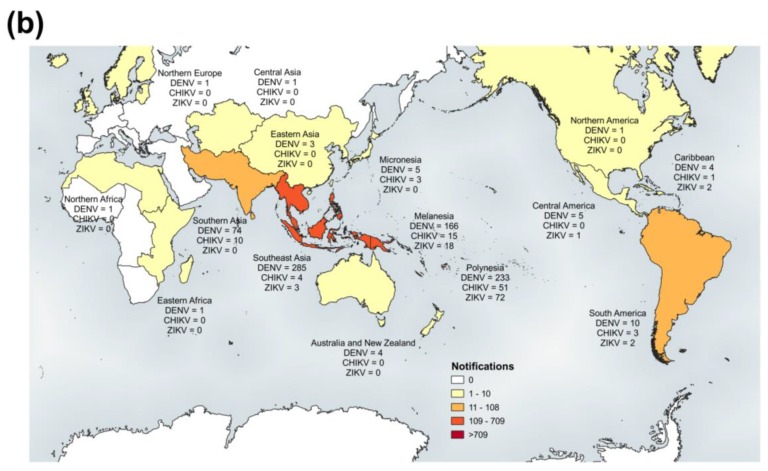
Notified cases among travellers to NZ by location of embarkation, 2008–2017. Subregional classifications were taken from the United Nations Statistics Division at https://unstats.un.org/unsd/methodology/m49/. (**a**) Number of notified confirmed DENV1–4, CHIKV, and ZIKV cases reported in NZ among travellers returning from the identified embarkation locations, 2008–2017 [70]. Specific locations or broader regional classifications were dependent on the travel history information available. All cases had travelled overseas; (**b**) Choropleth map of the number of positive notifications reported by ESR (2008–2017) based on subregional classifications. Map was created using QGIS software version 3.0.1.

The number of confirmed DENV 1–4, CHIKV, and ZIKV cases reported in NZ for the period, 2008–2017, by year and location [70] is summarised in Figure 7 and Figure 8, respectively.

A choropleth map summarizing the collective numbers of DENV-, CHIKV-, and ZIKV-positive notifications among travellers based on global subregional groups is shown in Figure 8b. Similar to the choropleth map showing RT-rtPCR/RT-PCR-positive samples reported by PHV (Figure 4b), most notifications were from infected travellers from the Pacific and Southeast Asia. In contrast to PHV, ESR reported cases from northern Africa, northern Europe, Central and East Asia, and North America. The one DENV case reported from northern Europe was unusual, given that DENV mosquito vectors have not been reported in this region and infection in this location was unlikely. Importantly, travel data provided by PHUs to reference laboratories is collated as accurately as possible; however, it is subject to patient recall, including symptom onset date. Whilst northern Europe was determined to be the probable location of infection based on patient data, the patient had also later travelled to Central Asia.

## 4. Discussion

The recent globalisation and co-circulation of the emerging arboviruses, DENV, CHIKV, and ZIKV, has been largely influenced by increased human movement and the unchecked spread of *Ae. aegypti* and *Ae. albopictus* mosquitoes. Improved testing and surveillance have also contributed to the increase in identification and reporting. Oceanic regions, like Australia, which host competent mosquitoes, are also largely inhabited by immunologically naïve human populations and are ideal environments for arbovirus emergence and disease [51]. Coupled with the complex dynamics of arbovirus evolution, limited availability and efficacy of vaccines, and the explosive rate of large-scale epidemics, it is recognised that no single strategy can completely solve such prevalent public health threats. The increasing demand on local medical laboratories to provide high through-put, rapid, and affordable diagnostics may also contribute to the associated economic costs to the community, where laboratories have passed on related costs to the patient. Whilst introduction of rapid, high through-put diagnostics often results in immediate, short-term benefits, sole focus on this alone, without employment of more robust, specialised diagnostics and expertise could jeopardize effective, long-term mitigation of these emerging pathogens or prevent detection of viruses that are co-circulating at a low level of transmission.

Local hospital and pathology laboratories perform an invaluable service and often provide frontline screening and diagnostic testing for arboviruses, such as DENV. Referral of samples from these entities to reference laboratories is a vital component belonging to the cascade of disease control strategies, which commence after potential cases are assessed by medical officers or following public health epidemiological investigations. Whilst it is important to recognise the necessity and enormous scale of the contributions made by local hospital and pathology laboratories and improve testing at the point of care, the resilience and adaptability of centralised reference laboratories is equally valuable for protecting our local, national, and state borders. Reference laboratories can intrinsically devote more resources to developing, curating, and performing a diverse, flexible range of specialised tests and often have research expertise to investigate complex disease issues. Within the Australasian region, there have been 4835 DENV, 171 CHIKV, and 149 ZIKV disease notifications collectively reported from Queensland (2005–2017) and NZ (2008–2017), underscoring the importance of specific viral diagnostics for these, often clinically similar, disease syndromes. Here, we have reviewed the reference testing performed by PHV and ESR, and the reservoir of specialist capabilities and public health support provided by these two laboratories. Timely reporting of confirmed positives directly to PHUs either by telephone, email, or integrated laboratory databases can expedite rapid disease and vector control strategies. This further supports the collaborative efforts of other multi-disciplinary agencies, including animal and environmental health departments, which are equally committed to a One Health approach for the overall improvement of global well-being [71]. 

Preparedness and early, rapid detection form the backbone of effective disease control and surveillance. This was evident during the recent onset and global surge of CHIKV and ZIKV activity when the availability of specific assays was limited. PHV responded to these challenges through foresight, utilisation of specialist expertise, and development of several in-house DENV, CHIKV, and ZIKV diagnostic assays, and continues to refine existing tests and explore new technologies, such as WGS and MIA serological platforms. Test evolution often requires in-depth monitoring of local and global strains over many years at both genetic and antigenic levels. Assays must remain relevant for currently circulating strains, which are often geographically (Figure 4 and Figure 8) and genetically diverse. Indeed, this afforded PHV the background data to re-design and upgrade their in-house DENV 1–4 RT-rtPCR assays for broader detection of circulating strains. Since 2014, ESR have also substantially increased and advanced their diagnostic and surveillance capabilities, with the introduction of DENV 1–4, CHIKV, and ZIKV molecular assays and CHIKV and ZIKV serology tests. 

The genetic relatedness among circulating arbovirus strains, as determined by nucleotide sequencing and molecular genotyping, plays a key role in disease management and can affirm epidemiological links and geographical outbreak sources. This is important as accurate travel history can be difficult for PHUs to obtain and may depend on patient availability for follow-up interviews, their memory, or a willingness to disclose related information. Phylogenetic analysis can further provide early warning of sustained transmission or show evidence of endemicity or hyperendemicity should it occur [18]. Nucleotide sequencing and genotyping have been revolutionized by WGS. PHV has introduced this technology for the surveillance and genetic characterisation of viral pathogens, including DENV, CHIKV, and ZIKV. Identifying potential geographical sources of viral strains and outbreaks is consequently more accurate due to the larger genetic dataset generated and allows a more robust analysis for the presence of potential pathogenic and genetic markers.

The full spectrum and diversity of specialised assays required to detect and provide timely awareness of co-circulating pathogens cannot be maintained without the collection, storage, and curation of archival reference isolates and patient sample material. Arbovirus collections are vital for phenotypic, genotypic, and evolutionary investigations and are instrumental for vector competence studies and the development of diagnostic assays, vaccines, and therapeutic agents [72]. PHV also contributes arbovirus isolates to the Royal College of Pathologists of Australasia for national quality assurance programs and to other requesting laboratories for the independent monitoring and scrutiny of laboratory assays and methodologies. Similarly, maintaining the capacity to isolate infectious DENVs could become essential for identifying antiviral resistance should antivirals be developed in the future and become widely used.

The inherent seriousness, high disease burden, and complex dispersal mechanisms associated with DENV, CHIKV, and ZIKV emergence and evolution warrants continued diligence and the availability of robust reference testing methods and surveillance. These specialised services, as outlined for PHV and ESR, are particularly vital in regions throughout Asia and the Pacific where developing countries are often directly burdened by arbovirus epidemics and may have limited resources to detect or control disease. As populations of *Ae. aegypti* and *Ae. albopictus* mosquitoes continue to increase and expand, and human travel escalates, a new global epidemic or emergence of a new arbovirus could be imminent. Thus, continuous improvement, advancement, and dissemination of our current knowledge and expertise, combined with the comprehensive efforts of local and specialised reference laboratories, are vital for ongoing limitation and mitigation of future disease threats.

## Figures and Tables

**Table 1:** RT-rtPCR assays for dengue virus (DENV), Zika virus (ZIKV), and chikungunya virus (CHIKV) currently used by the Public Health Virology Laboratory (PHV).

Virus	RT-rtPCR/RT-PCR *^a,b^*	Genome Region *^c,d,^*^e,*f*^
DENV 1–4	DU5 MGB2017 *^a^*	3′UTR
DENV-1	DENV-1 TM2017*^a^*	3′UTR
DENV-2	DENV-2 MGB*^a^*	3′UTR
DENV-3	DENV-3 TM2017*^a^*	3′UTR
DENV-4	DENV-4 TM2017*^a^*	3′UTR
DENV 1–4	DENV 1–4 nested *^b^* [34]	C-prM
ZIKV	ZIKV Pacific E*^a^* [11]	E
ZIKV	ZIKV Pacific NS1 *^a^* [11]	NS1
CHIKV	CHIKV MA *^a^* [35]	E1

*^a^* RT-rtPCR assay; *^b^* RT-PCR assay; *^c^* 3′ untranslated region (3′UTR); *^d^* capsid-premembrane (C-prM); *^e^* envelope (E); ***^f^*** envelope 1 (E1).

**Table 2:** Number of notifications of DENV, CHIKV, and ZIKV reported per year from Queensland (2005–2017) and New Zealand (NZ) (2008–2017).

	Queensland			NZ		
Virus	Years Reported *^a^*	Total *^b^*	Mean *^c^*	Years Reported *^a^*	Total *^b^*	Mean *^c^*
DENV	2005–2017 (13)	4037	311	2008–2017 (10)	798	80
CHIKV	2008–2017 (10)	84	8	2011–2017 (7)	87	12
ZIKV	2014–2017 (4)	51	13	2014–2017 (4)	98	25

*^a^* Years in which notifications were reported, with total number of years in brackets. *^b^* Total number of notifications for the years reported. One additional case of sexually transmitted ZIKV was also reported from NZ [62]. *^c^* Mean number of notifications per year calculated from the total number of notifications and total number of years in the reporting period.

## Supplementary Materials

The following are available online at http://www.mdpi.com/2414-6366/3/3/75/s1. Figure S1. Testing algorithms for acute DENV infections performed by PHV. Figure S2. RT-rtPCR-positives reported by PHV for CHIKV (2008-2017) and ZIKV (2014-2017). Figure S3. Number of CHIKV isolates obtained, PHV, 2008-2017. Figure S4. Northern Queensland DENV outbreaks, 1990-2017. Table S1. Oligonucleotide primer and probe sequences and viral gene target regions for PHV pan-DENV, DENV 1-4, CHIKV and ZIKV RT-rtPCR assays. Table S2. The number of notified DENV 1-4, CHIKV and ZIKV cases reported in NZ for the period 2008-2017 by location (region travelled from or residence).
