# Erratum: Pyke, A.T. et al. On the Home Front: Specialized Reference Testing for Dengue in the Australasian Region. *Trop. Med. Infect. Dis.* 2018, *3*, 75

**DOI:** 10.3390/tropicalmed4040129

**Published:** 2019-10-16

**Authors:** Alyssa T. Pyke, Wendy Gunn, Carmel Taylor, Ian M. Mackay, Jamie McMahon, Lauren Jelley, Ben Waite, Fiona May

**Affiliations:** 1 Public Health Virology Laboratory, Forensic and Scientific Services, Coopers Plains, QLD 4108, Australia; Carmel.Taylor@health.qld.gov.au (C.T.); or Jamie.McMahon@health.qld.gov.au (J.M.); 2 Institute of Environmental Science and Research Limited, Wallaceville, 5018 Upper Hutt, New Zealand; wendy.gunn@esr.cri.nz (W.G.); Lauren.Jelley@esr.cri.nz (L.J.); Ben.Waite@esr.cri.nz (B.W.); 3 Child Health Research Centre, The University of Queensland, South Brisbane, QLD 4101, Australia; 4 Metro North Public Health Unit, Metro North Hospital and Health Service, Queensland Health, Windsor, QLD 4030, Australia; Fiona.May@health.qld.gov.au

The authors wish to make the following correction to Table 1 of this paper [1]:

The DENV 1–4 DU5 MGB2017 real-time polymerase chain reaction (RT-rtPCR) assay should state that it is designed in the Capsid peptide coding region under the genome region column, and not the 3’UTR.

This change has no material impact upon the conclusions of our paper. The authors would like to apologize for any inconvenience caused to the readers by this change.

## Figures and Tables

**Table 1:** RT-rtPCR assays for dengue virus (DENV), Zika virus (ZIKV), and chikungunya virus (CHIKV) currently used by the Public Health Virology Laboratory (PHV).

Virus	RT-rtPCR/RT-PCR *^a,b^*	Genome Region *^c,d,e,f^*
DENV 1–4	DU5 MGB2017 *^a^*	Capsid peptide coding region
DENV-1	DENV-1 TM2017 *^a^*	3′UTR
DENV-2	DENV-2 MGB *^a^*	3′UTR
DENV-3	DENV-3 TM2017 *^a^*	3′UTR
DENV-4	DENV-4 TM2017 *^a^*	3′UTR
DENV 1–4	DENV 1–4 nested *^b^* [34]	C-prM
ZIKV	ZIKV Pacific E *^a^* [11]	E
ZIKV	ZIKV Pacific NS1 *^a^* [11]	NS1
CHIKV	CHIKV MA *^a^* [35]	E1

*^a^* RT-rtPCR assay; *^b^* RT-PCR assay; *^c^* 3′ untranslated region (3′UTR); *^d^* capsid-premembrane (C-prM); *^e^* envelope (E); *^f^* envelope 1 (E1).
