# Supplementary material for: On the Home Front: Specialised Reference Testing for Dengue in the Australasian Region
Source: Trop Med Infect Dis. 2018 Jul 15;3(3):75. doi: 10.3390/tropicalmed3030075 (PMC6161173; doi:10.3390/tropicalmed3030075)
Supplement: Supplementary file 1 [file tropicalmed-03-00075-s001.zip › tropicalmed-315747-supple fig Proof.docx]

**Supplementary Materials:**

1. The following are available online at [www.mdpi.com/xxx/s1](http://www.mdpi.com/xxx/s1),


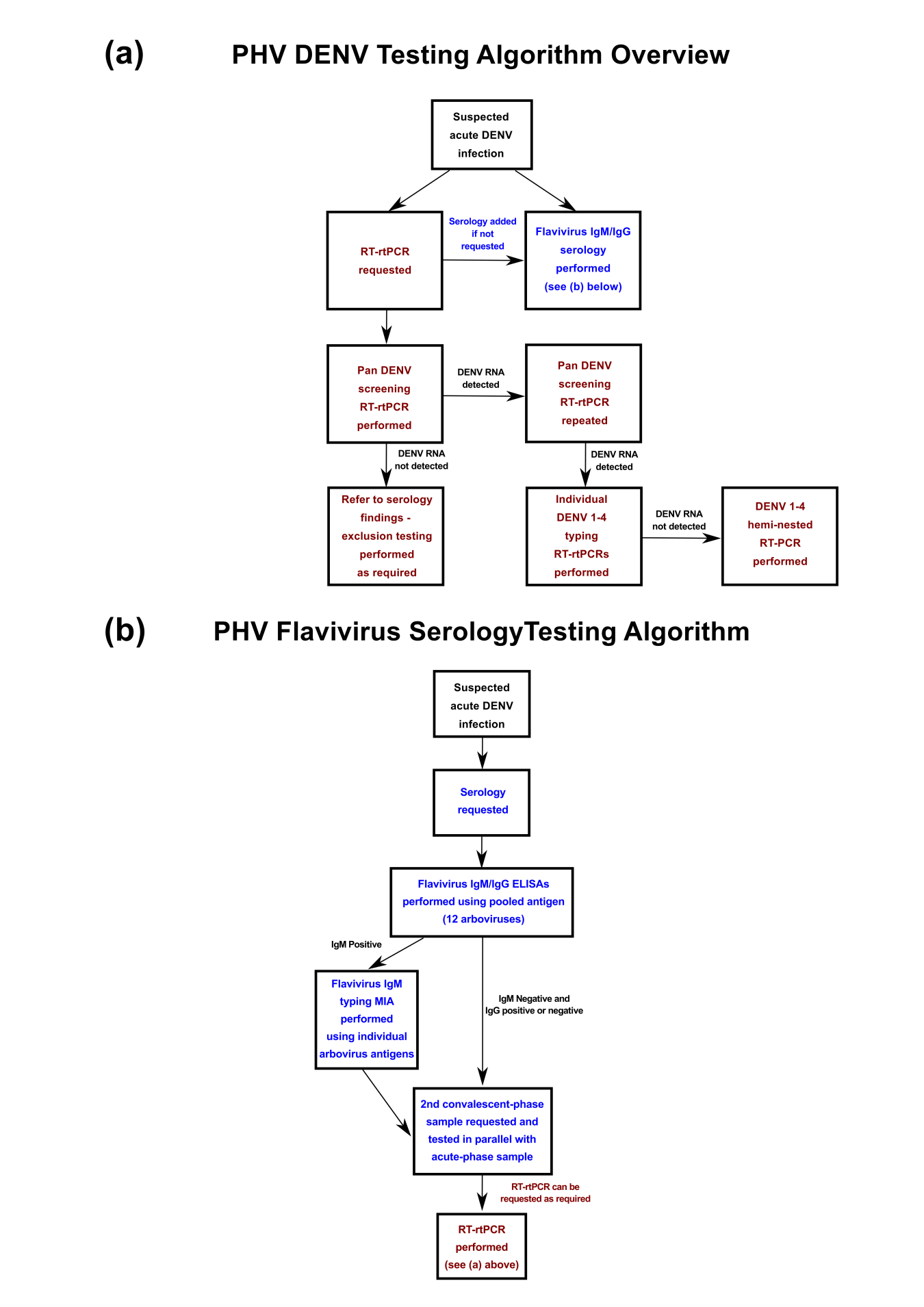
**Supplementary Figure 1. Testing algorithms for acute DENV infections performed by PHV.** (a) Overview of molecular (RT-rtPCR/RT/PCR) and serological testing. (b) Detailed summary of serological testing.


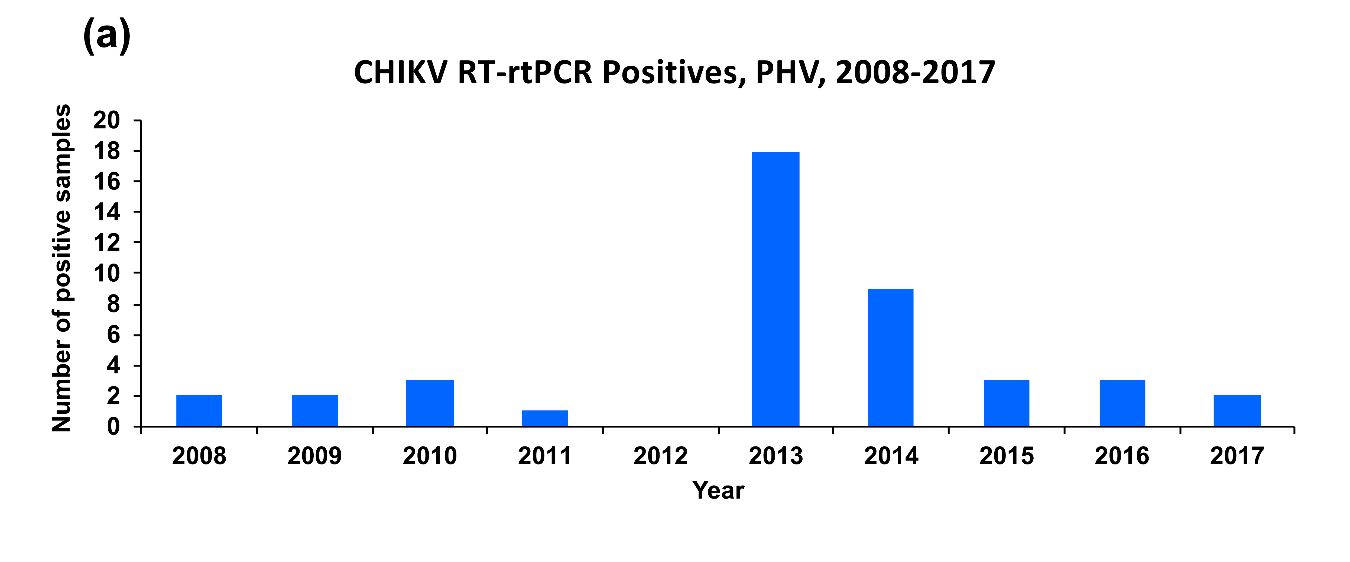


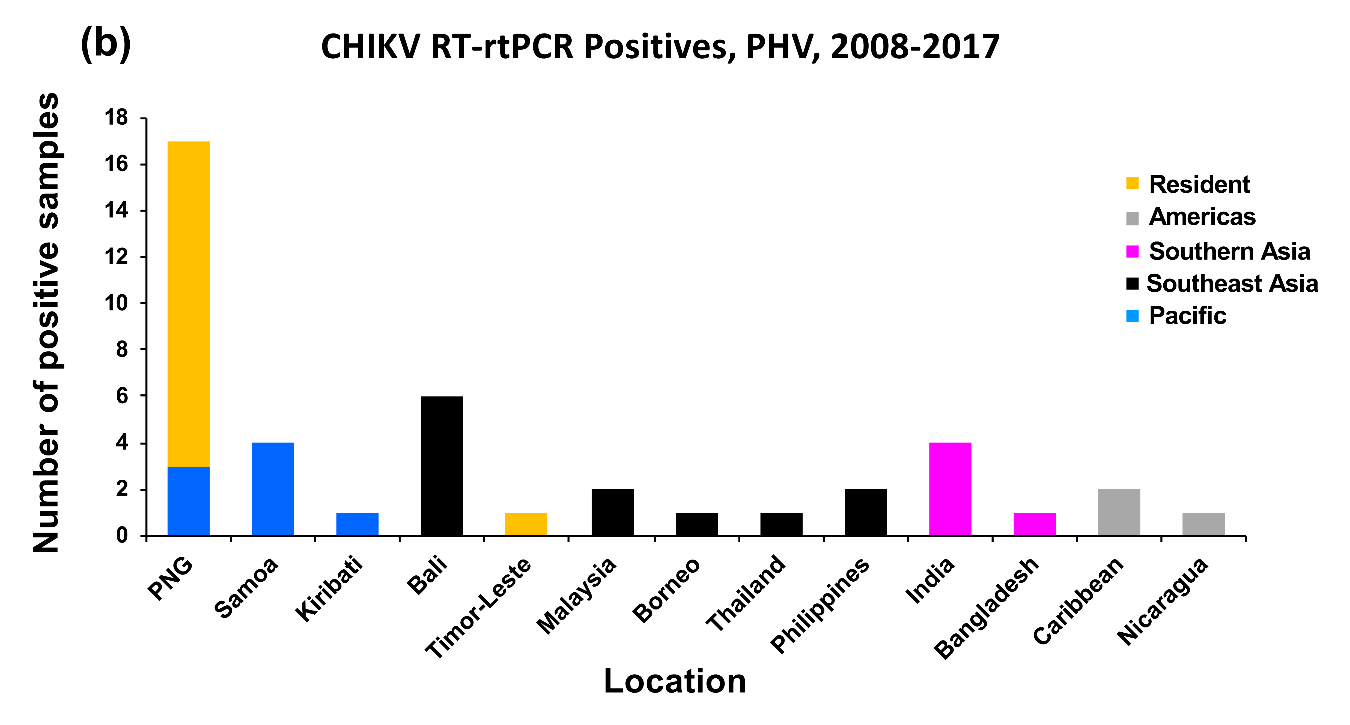


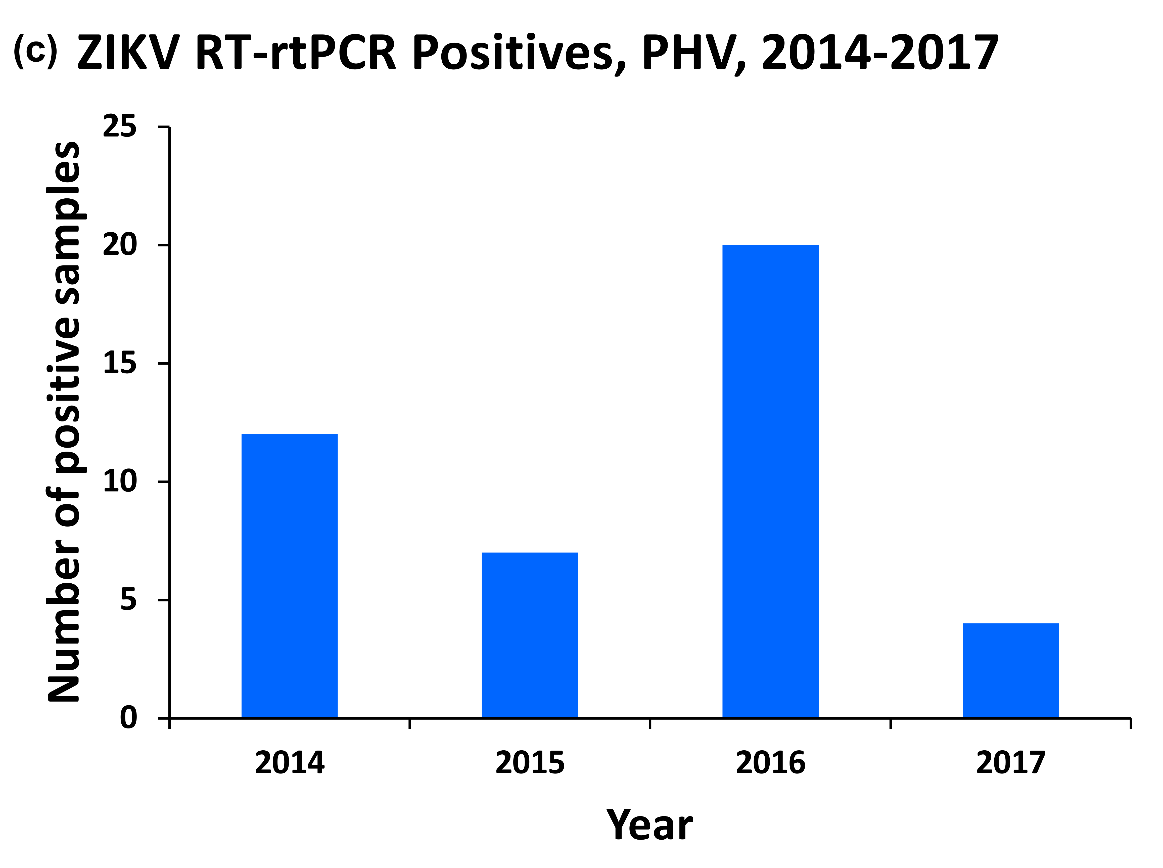


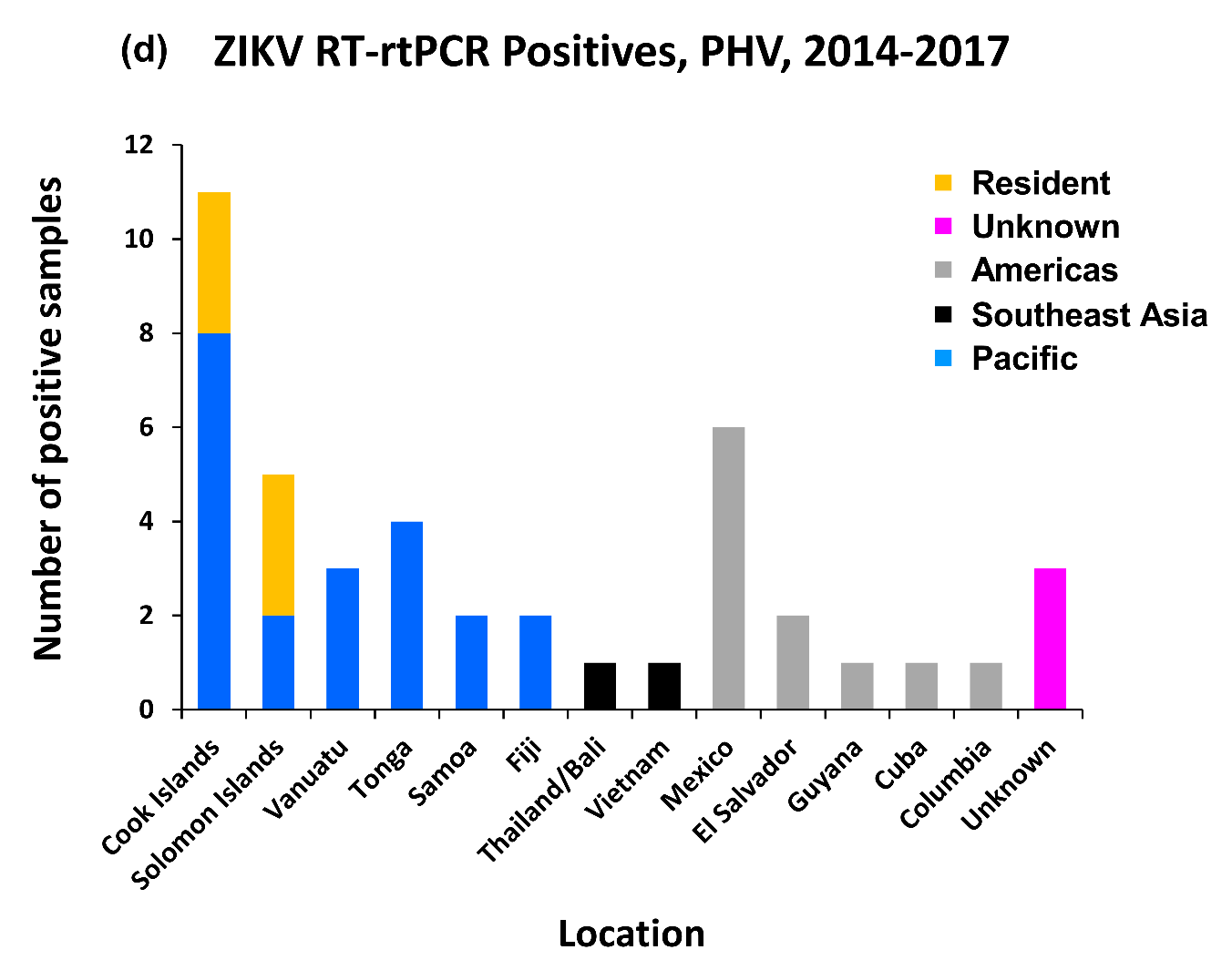


**Supplementary Figure 2. RT-rtPCR positives reported by PHV for CHIKV (2008-2017) and ZIKV (2014-2017).** Number of CHIKV RT-rtPCR positives by (a) year and (b) location (region traveled from or residence). Number of ZIKV RT-rtPCR positives by (c) year and (d) location (region traveled from or residence).


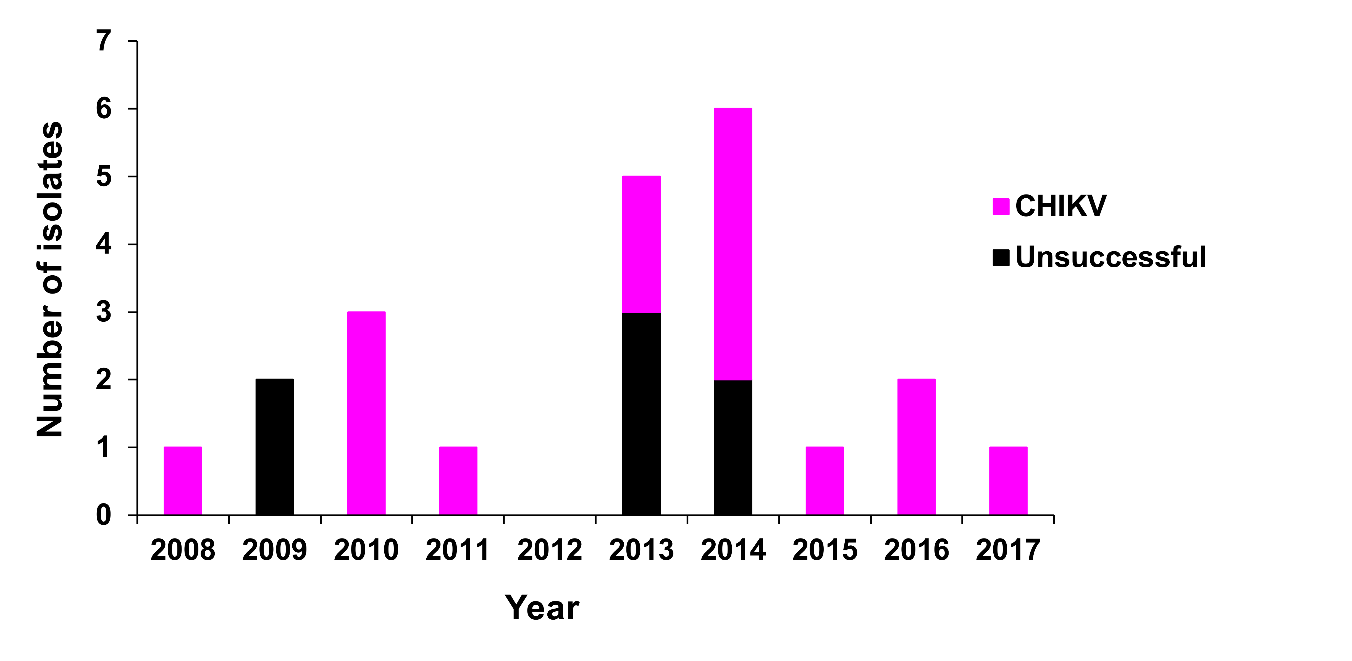


**Supplementary Figure 3. Number of CHIKV isolates obtained, PHV, 2008-2017.** The number of RT-rtPCR positive samples from which CHIKV isolates were obtained (pink bar) or not obtained (black bar) is highlighted per year.


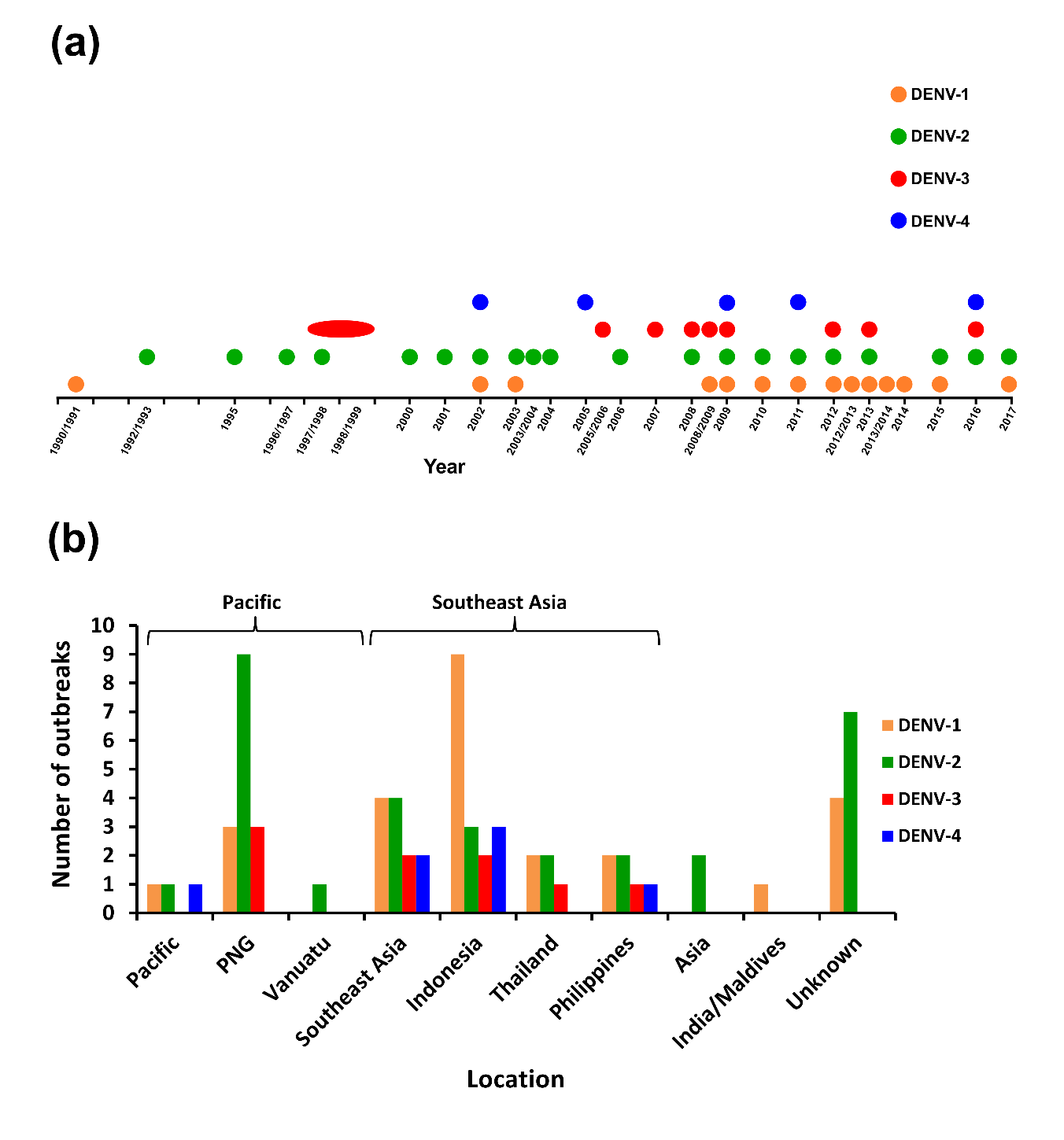


**Supplementary Figure 4. Northern Queensland DENV outbreaks, 1990-2017 (https://doi.org/10.6084/m9.figshare.6193073.v3).** Figure adapted and used with permission, Microbiology Australia ( Pyke, A. T.; The origins of dengue outbreaks in northern Queensland, Australia, 1990-2017. *Microbiol. Aus.* **2018**, 39. Doi: 10.1071/MA18027). Data includes secondary outbreaks which resulted from further spread of imported DENV strains into new Queensland locations. (a) A timeline summarizing the occurrence of northern Queensland outbreaks between 1990 and 2017 based on circulating DENV 1-4 serotypes. The sustained DENV-3 outbreak in 1997-1999 is represented by an elongated ellipse symbol. (b) The number of northern Queensland outbreaks occurring between 1990 and 2017 are plotted against the location (region traveled from or residence, given as specific country or broader regional classification where country data was unavailable) of DENV RT-rtPCR/RT-PCR positive patients. The most likely geographical source of outbreaks was inferred following phylogenetic analysis.

**PHV ELISA Protocols**

Patient serum is reacted in flavivirus IgG and IgM-MAC ELISAs against a pool of flavivirus antigens, including DENV 1-4, Japanese encephalitis virus (JEV), Murray Valley encephalitis virus (MVEV), Kunjin virus (KUNV), Alfuy virus (ALFV), Kokobera virus (KOKV), Stratford virus (STRV), yellow fever virus (YFV) and ZIKV. To prepare the pooled antigen, each virus is diluted to give equivalent reactivities which have been pre-determined using a monoclonal antigen capture and detection ELISA. An equivalent negative control pool is prepared using the host cell lines in which each virus was grown.

**Flavivirus IgG Antigen-capture ELISA:**

Plates are coated with anti-flavivirus monoclonal antibody WR/DEN2/4G2 (Cell labs, Australia). Virus and control pool antigens are added to alternate columns of the plate, incubated for 1 hr at 37°C before washing with PBS-Tween. Test and control sera are diluted 1:200 in milk diluent prepared from Milk Diluent/Blocking Solution Concentrate (Kirkegaard & Perry Laboratories, USA) before addition to the plate in 50 µL volumes. The plate is then incubated and washed as previously before the addition of 50 µL per well HRP-labelled anti-human IgG (DakoCytomaton) followed by incubation and washing as previously. TMB substrate (Elisa Systems, Australia) is added to each well followed by incubation at 37°C for 10 minutes after which 50 µL 1N H_2_SO_4_ is added to each well to stop the reaction. Plates are then read at an absorbance 450/620 nm on a plate reader.

**Flavivirus IgM MAC-ELISA:**

Plates are coated with Rabbit antihuman IgM Mu chains (Dako). Test and control sera are diluted 1:100 as for the IgG ELISA, and added to the plate followed by incubation and washing as above. Antigen and control pools are then added to alternate columns followed by incubation and washing. Bound antigen is detected by addition of 50 µL per well 6B6C-1 monoclonal antibody (Merck) conjugated to horse radish peroxidase (HRP) (prepared in-house). TMB substrate (Elisa Systems, Australia) is added to each well, followed by incubation at 37°C for 10 minutes. The reactions are stopped by adding 50 µL 1N H_2_SO_4_ per well before plates are read at an absorbance of 450/620 nm on a plate reader.

**Table S1.** Oligonucleotide primer and probe sequences and viral gene target regions for PHV pan-DENV, DENV 1-4, CHIKV and ZIKV RT-rtPCR assays.

**Table S2.** The number of notified DENV 1-4, CHIKV and ZIKV cases reported in NZ for the period 2008-2017 by location (region traveled from or residence).
